# Collectanea Medica

**Published:** 1812-03

**Authors:** 


### COLLECTANEA MEDICA,

CONSISTING OF

### ANECDOTES, FACTS, EXTRACTS, ILLUSTRATIONS, QUERIES, SUGGESTIONS, &c.

RELATING TO THE

*History or the Art of Medicine, and the Auxiliary Sciences.*

---

### *Observations on the Natural and Medical History of the Zetland Sheep.—By* Dr. A. Edmonston.

THERE is no animal more generally useful to man than the sheep. Its gentle disposition, the facility with which it can be tamed, the delicacy of its flesh, and the richness of its covering, early rendered it essential to human society; and in the progress of arts it constituted a principal source of the wealth of nations. The management of sheep, the improvement of the different races, and the cure of their diseases, have, therefore, become pursuits of general interest; and every fact which tends to illustrate these subjects naturally claims our attention.

It is while uninfluenced by the effects of domestication, that we can best observe those instincts which guide animals, and trace the operation of external powers. As the sheep in the Zetland islands, instead of being under the control of shepherds, are permitted to range according to their inclination, they exhibit in their modes of life the natural propensities of the species; and we can therefore, in that country, attain a knowledge of some of the peculiarities which actuate the race.

The native Zetland sheep is very small, compared with those of the southern parts of Scotland and England, the carcase not weighing more on an average than 30lbs. It is of a handsome shape, hardy, and very swift and agile. The general length, from the tip of the nose to the root of the tail, is about 38 inches; and the height, from the top of the shoulder to the sole of the foot, 14 inches. The tail seldom exceeds 3 inches in length. The horns are small, and the ears stand erect. The ram has four large and beautiful horns: the upper pair gradually diverge a little backwards, and then curve in towards the ears and sides of the head, in a spiral form, somewhat like those on the head of the black-faced heath ram of Yorkshire; the lower pair are nearly of a semicircular shape, with the extremities almost meeting under the throat.

A breed of sheep exactly similar to that in Zetland, appears pears to exist in Iceland (Von Troil's Letters, 136), and in the Faro islands; and it is probable, that both have sprung from the same source, and that similar causes and modes of management have continued to preserve the identity between them.

The Zetland sheep are of different colors, as white, grey, black, speckled, and a dusky brown, called moorit. The face is generally of the same color with the body, and is somewhat shorter, in proportion, than among the sheep of Scotland. The prevailing color is white, with but little admixture; the next is the grey. Some are almost entirely black, with only a small portion of white above one or more of the hoofs. More frequently the forehead, face, and legs, and a part of one thigh, are white, and the rest of the body perfectly black. There is nothing of that uniformity of color among them, corresponding to what takes place in the black-faced sheep of this country.

The quality of the wool varies very much on the same animal; the thighs and back yielding the coarsest, and the neck and breast the finest, wool. Stockings have been made of the wool from 5s. to 30s. per pair; and it is by no means uncommon to obtain the materials from which both are manufactured, from the same animal. Generally speaking, softness and fineness are characteristic properties of Zetland wool, in which respect it is not surpassed even by the Merino wool.

The ewes yean in the months of April and May, and a few even in March, and they generally drop one lamb each. It is by no means uncommon for an ewe to drop two lambs, and some three. In most parts of the country, the lambs suck as long as their mothers permit them, which is between three and four months. In those districts where it is considered an object to milk the ewes, the lambs are gagged for several hours to prevent them from sucking. This operation is called haveling the lambs.

In Zetland, almost all the sheep run wild on the hills, and they appear to be fond of climbing like goats upon the rocks, in search of rich tufts of grass. Many suffer from this practice, by venturing on places from which they cannot return. In winter, the arable land is intentionally thrown open to their incursions; but in summer and autumn, every effort is practised to exclude them from the inclosures. As the dikes, however, are low and ill-built, the sheep frequently leap over them, and often seriously injure the crop; and in some parts of the country it is the custom to chace them from the neighbourhood of the farms by dogs, every night at sunset, while the corn is on the ground. Although large tracts of pasture are common to several farms, yet the sheep which belong to each seldom stray far beyond their proper limits, and if transported to a distant district, frequently return to the spot of their nativity.

The different owners know the particular individuals which belong to them by a mark on the ear, and the people can discriminate slight differences of this kind with wonderful accuracy, even at a great distance. In summer they are driven into a small circular inclosure called a crue, for the purpose of taking off the wool. The native sheep are seldom shorn; but about the beginning of June, when the fleece begins to loosen spontaneously, it is pulled off by the hand. This operation is called rooing the sheep. They are left very bare after it; but the people say, that the wool on the animal continues much finer when it is removed in this manner, than by the shears. Except for the purposes of rooing, milking, raveling, and marking, them, on all other occasions the sheep are caught by dogs trained for the purpose. It is surprising to see with what accuracy a dog singles out the particular sheep from among a flock; and in a short time the individuals who are not the objects of pursuit, appear to be sensible of the election in their favor, and become comparatively tranquil while the other is hunted down.

Although thus early accustomed to view the dog as an enemy, they readily associate together; for if a lamb happens to be reared in the house, in consequence of the early death of the mother, it plays and even fights with dogs, and readily joins them in the pursuit of other animals.

The sheep are never taken under cover in the winter time; nor, in case of snow, is there any food provided for them. On this latter account they suffer greatly, having little else to feed upon, for several weeks in succession, but the sea-weed growing on the shores, or what has been drifted on the beach by the surf. It is curious to observe with what precision they leave the hills, and betake themselves to the sea-side, at the moment the tide of ebb commences. This I can state to be an absolute fact, although I am utterly unable to explain by what process of sensation or of instinct it is effected. From remaining quiet on the hills, and endeavoring to browse on their summits, a whole flock is seen suddenly to run towards the sea-shore, and, on observing the state of the tide, it is found that the water has just begun to recede.

On the coming on of a storm of snow, they retire to the more sheltered places, which are generally in the neighbourhood of the sea. There they are frequently snown over,

 and, and, by the subsequent melting and falling down of the snow, are sometimes thrown over the rocks into the sea. On such occasions they frequently assemble in considerable numbers on the side of a hill, and place themselves in such a manner as that their heads all incline towards the centre. By this arrangement their breath keeps them warm, and, dissolving part of their icy covering, forms a kind of vault over their heads. In this situation they have been known to remain for many days, during which they appear to maintain life by eating the wool from off each other's backs.

Such a mode of managing the sheep no doubt renders them obnoxious to many diseases, and it is certainly an ungrateful return to those who so often have

> —— given us milk in luscious streams,
> And lent us their own coat against the winter's cold.

Yet, except in seasons when the weather is unusually bad, and the food scanty, they may be said to be comparatively healthy; and the most severe and fatal distempers with which they are afflicted, have been imported into the country within a period of forty years.

Blindness first made its appearance among the Zetland sheep in 1770, and it was traced to communication with a ram from Montrose, which labored under the disease at the time he was brought to the country. This affection consists in the formation of a film over the whole anterior part of the cornea, which produces complete blindness. When the attack is not very violent, it wears off in about a fortnight, and it is observed to disappear more readily when the animal is left to run at large, than when taken into a house; but if not protected, it suffers greatly by falling into mossy pits, and over rocks, and from the attacks of birds of prey.

Mr. Stevenson, the Rev. Mr. Singers, and Mr. Hog,* have each assigned causes for this affection, which, as applied to the production of blindness in Zetland, appear to me to be unsatisfactory.

Mr. Stevenson ascribes it to the reflection of heat and light, in very sunny and dry weather, as it is observed, he says, to be more frequent when the hills become scorched, and on hard rocky soils, than on dark-colored hills, covered with heath. The parish of Delting, into which the disease was first introduced, consists almost entirely of dark-colored

* A Treatise on the Diseases of Sheep, drawn up from original communications, presented to the Highland Society of Scotland, by Andrew Duncan, jun. M.D.—*Transactions of the Highland Society*, vol. iii.

mossy hills; and this topographical fact, coupled with the general dampness of the climate of Zetland, tends to shew that the exciting causes mentioned by Mr. Stevenson, never had operated where the disease raged with its greatest malignity.

The Rev. Mr. Singers conceives, that this affection may be produced sometimes by the pollen of flowers irritating the eyes of the sheep, when blown in considerable quantity. In the hilly pasture of Zetland, there is not the appearance of a flower in the course of many miles; and the few which do yield pollen, occur in the inclosures, from which the sheep are carefully excluded during the summer.

Mr. Hog ascribes blindness to continued fatigue and exertion; and endeavors to illustrate his opinion by the examples of the affection occurring in a severe degree in sheep that have been driven a long distance to market. In the Zetland islands, sheep are never driven more than five or six miles at a time, and that only with a view to assembling them in a crue, for the purposes already mentioned. They are driven in the gentlest manner on such occasions, and not a single instance of blindness has ever been known to ensue from this treatment.

That the different exciting causes enumerated by these gentlemen, when applied in sufficient force, may produce inflammation of the eyes, I have little doubt; but they must be considered as being purely local and adventitious, and can never be supposed to exert an influence beyond the scene of their immediate action. In the Zetland islands they are not known to operate, and yet a general disease exists, which exhibits, in its commencement and progress, a train of symptoms similar to that which characterises the affection which has been ascribed to their presence.

These considerations lead me to conclude, that the belief in Zetland, that blindness was originally imported into the country, and is at present propagated by contagion, is strictly correct. This opinion derives strength from different circumstances. The period of the first appearance of the disease is fresh within the memory of many, and the fact is related by persons of unquestionable veracity and accuracy of observation; and it has been found to spread itself from the place where it first occurred, as from a centre. There are several parts of Zetland into which it has never yet found admission, although separated from those in which it rages, by a narrow ferry only. This fact supports the idea of a contagious principle. Were the disease in general the offspring of external causes, occasional instances would certainly be produced, although neither so frequent nor so numerous as in situations where their presence is more uniform.

From a general view of the usual progress of blindness in sheep, as mentioned by different writers on the subject, I am disposed to believe, that the pathology of the different affections is not always well understood. In some cases there is genuine ophthalmia, when the inflammation extends from the conjunctiva all over the lucid cornea. This is among the worst species of the affection, and it requires the most energetic treatment. But in those instances, where "a blue slough covers the whole of the eye, without any admixture of red vessels," as mentioned by Mr. Stevenson, I am inclined to believe, that inflammation had never existed. It is not a general quality of inflammation of the eye, which has proceeded the length of forming preternatural membranes, to subside spontaneously in so short a time as a fortnight, nor to be more readily removed when the individual labouring under it is exposed to light and air, than when secluded from their influence.

In some of those lighter attacks, where a blue film appears to cover the cornea, I presume that the appearance is occasioned by the mere distention of the cornea, from an increase in the quantity of the aqueous humor. This will produce a perfect opacity in the membrane, (see a Treatise on the Varieties and Consequences of Ophthalmia, p. 173.) It appears to be produced in the human species and in horses and dogs, by whatever tends to accelerate the motion of the blood to the head; and analogy leads me to think that it may also be a frequent cause of temporary blindness among sheep. It is best removed by a change of pasture, and repeated doses of purgative medicines.

The scab was first introduced into the parish of Dunrossness, the most southern district in the country, about twenty-four years ago, by two lambs sent from Scotland as a present to a gentleman, with a view of improving the breed of sheep in Zetland. The circumstances, which favored the propagation of the disease over the country, are curious and authentic. While the two infected lambs remained in a shed, the gentleman proposed sending a sheep of the Zetland breed to a friend in Lerwick. One was accordingly taken from the hill, bound and ready to be put into a boat; when, unfavorable weather coming on, the passage was delayed for several days. In the mean time the native sheep was put into the same place with the foreign lambs, and communicated with them. When the weather became favorable for the departure of the boat, the sheep intended to have been sent by it was found to have suffered so much by confinement confinement that it was not thought sufficiently good, and was returned to the hill and another taken in its stead. Soon after this, the scab, the presence of which had never been suspected, broke out on the two lambs. They were immediately killed, but the reprieved sheep had already imparted the fatal present to a whole flock; and this disgusting disease has been extending its ravages ever since.

The destructive effects of the scab have been very obvious. Many individuals who had four or five hundred sheep a few years ago, have not now more than half a dozen, and the affection is no doubt heightened by the want of proper remedies for its removal, and the careless management of those affected by it. As the whole sheep of the country run wild among the hills, a single individual may affect multitudes; and the disease, once induced, is aggravated by exposure to cold, damp, and scarcity of food.

> Turpis oves tentat scabies, ubi frigidus imber
> Altius ad vivium persedit, et horrida cano
> Bruma gelu.*

Dry pasture and the common mercurial ointment of the shops, are the best remedies for its removal. The ointment should be rubbed on the affected parts and be well incorporated with the skin.

Inflammation of the stomach and bowels, is a frequent affection among the sheep in Zetland. It is known there by the name of vinster sickness. From the suddenness of its invasion, the ambiguity of the symptoms, and the rapidity of their progress, it is generally fatal; and, as the mortified part exhibits a black appearance, the people conceive it to be produced by the rupture of a blood-vessel. The slighter degrees of this disease frequently lay the foundation of obstinate dysentery.

Water-sickness, a general dropsy, is also a frequent disease among the sheep. It occurs under the different forms of anasarca, or water in the cellular substance immediately under the skin, and ascites, or water, in the abdomen. This last species of the disease takes place most commonly in wet autumns, continues during the greater part of winter, and sometimes carries off whole flocks. Tapping has been tried, but I believe never with success.

The sturdy, or dropsy in the brain, is a very common and fatal disease. The operation of trepanning the skull, and

* Georgic, lib. iii. ver. 441.—This observation of the poet, shews the antiquity of the disease, and the aggravation of the symptoms by exposure to cold and moisture.

extracting the water-bag, which lies upon the brain, has been repeatedly and successfully performed since 1778, by individuals, who never either saw or heard of a treatise on the diseases of sheep. A similar operation has also been successfully performed on the cow.

The disease known in Zetland by the name of the shell-sickness, consists in a thickening and concreting of the omentum and larger intestines into small white lumps resembling shells, from which it derives its name. It is common to sheep which feed on wet mossy pastures. They get lean, are disinclined to move much about, and the belly feels unequally hard. The people drive the sheep, when affected with this complaint, to the sea-side, and force them to eat sea-weed, and swallow salt-water, as the only cure. These substances operate as purgatives, and may, by that means, contribute to remove the complaint.

The rot, known in Zetland by the name of mua-sickness, is also one of the diseases with which the sheep are frequently affected. Various opinions have been entertained respecting the cause and seat of this disorder. Dr. Coventry, with great propriety, conceives, that the most common species of it arises from deficient and depraved aliment, and that it somewhat resembles the scurvy in the human species.* I am persuaded, that this view of the subject is correct, as applied to Zetland, where the causes assigned for its production so often exist, and I am inclined to believe, that the affection of the liver should be viewed rather in the light of a secondary symptom. The general debility induced by the combined effects of cold, moisture, and depraved aliment, lays the foundations of glandular obstructions and dropsical affections. The liver suffers with the other organs, and becomes the seat of a peculiar species of hydatid, in which an insect (*fasciola hepatica*†) is usually contained; but the presence of the insect in the liver, although a frequent concomitant of the rot, is not absolutely essential to its existence.

Removal of the diseased individuals to dry situations, nutritious food, and the occasional use of mercurial purgatives, are the remedies chiefly to be trusted to; and if employed in the early stages of the disease, seldom fail to effect its cure.

As the other diseases with which the Zetland sheep are

---

* Introductory Discourse.

† This insect is of a flat circular form, and often three quarters of an inch in diameter. It flaps vigorously on a table, when removed from its nidus.

affected, affected, present nothing peculiar, either in their progress or consequences, it is unnecessary to enumerate them.—*Memoirs of the Wernerian Natural History Society.*

---

*Report made to the Institute upon a Memoir of M. Tarry, on the Composition of Writing Inks.*

Messrs. Berthollet, Vanquelin, and Deyeux, being appointed by the class to give an account of the memoir of M. Tarry, M.D., M. Deyeux drew up the following relation.

The author of the memoir professes to point out, 1st, The methods employed to make characters disappear from the paper on which they are written; 2d, The means of restoring writing which to all appearance had been destroyed; 3d, The means of perfecting common inks; 4th, The discovery of an ink which will resist all chemical agents.

Article I.

The art of erasing writing is very ancient, and the means of effecting it are very easy. It is well known that we have only to moisten the paper with any acid, to cause the writing to become less perceptible, and by degrees to disappear altogether. But all acids cannot be used with the same success; some leave a mark on the paper which is not easily removed, and others corrode the paper so as to render it unfit for future use. To avoid these inconveniences, it is requisite to use an acid which has only an effect on the writing, and not on the paper, and which will not change the color of it.

To ascertain this point, the author has subjected common inks to the action of different acids, and has carefully observed the phenomena which resulted from the mixture of these different bodies. According to him, sulphuric acid readily destroys the writing, but at the same time it gives the paper an oily tinge. The oxalate acid of potash produces more prompt and certain effects. The oxigenated muriatic acid, if fresh, is preferable to the two former; for, at the same time that it destroys the writing, it whitens the paper without otherwise altering it. This is not the case with the nitric acid, which, though it always destroys the writing, penetrates into the paper, and leaves waving yellow lines. These inconveniences, however, may in some degree be remedied, by diluting the acid with water, or by dipping the paper in this fluid as soon as the writing has disappeared. A mixture of nitric and of muriatic acid, acts but slowly on the writing. It whitens the paper, and does not oppose its drying, as is the case with the nitric acid. In general, whatever acid is used, it is necessary to immerge the paper in water, water, to dissolve the new combinations which the acids form with the parts of the ink which have been taken away.

M. Tarry observes, that the China ink is not acted upon by acids like common ink, its composition being very different. Instead of destroying the writing, acids render this ink of a darker color, and they can only be destroyed by a knife.

Article II.

*Process for ascertaining the Writing which has been substituted for that which was removed, and means of restoring that which had been destroyed.*—The means that have been indicated of destroying writing, depend, as has been observed, on decomposing the ink, and making its constituent parts form new combinations. These combinations, by being decomposed in their turn by different agents, recover a color, which, if not that of ink, yet presents one which is sufficiently evident to enable us to discover the letters and words that had been traced on the paper before the acids had been applied to it.

According to the author, the gallic acid is one of the agents which in this case succeed pretty well. The liquid precipitate of lime also has a good effect. So also have the alcaline hydrogened sulphurs. But these agents never succeed if the acids have remained long on the writing, and if care has been taken to dip the paper in water. It may easily be conceived, that in this case the constituent parts of the ink which were combined with the acid, and had formed with it combinations soluble in water, being taken away by this fluid, they ought not to leave any traces of their existence, and that consequently it is impossible that the agents used to discover them can render them visible. For this reason, the gallic acid, the liquid precipitate of lime, the alcaline hydrogened sulphurs, and many other re-agents so much boasted of, ought no longer to be regarded as infallible means of causing the writing to re-appear.

Article III.

*Of perfecting common Inks.*—It is of great consequence that the inks of commerce should be of good quality. Some are destroyed spontaneously; others gradually lose their black color, and become yellow; some penetrate the paper and alter its nature; whilst others become darker with time. All these effects are produced by the different substances employed in the composition of the inks.

Convinced of the advantage of having good ink, the author has made many experiments to obtain one of superior quality. But after all his researches, he has found none better than that of M. Lewis, of which the receipt has been

 published.

published. That ink, according to him, has all the perfection that can be desired, and therefore it should be generally adopted; but at the same time we ought to state, that it is as capable as the others of being dissolved by acids, and those who have an interest in making writing disappear, will know how to avail themselves of this circumstance.

For this reason, no doubt, M. Tarry has thought himself obliged to make new experiments to obtain an ink unalterable by chemical agents. To arrive at the discovery of this perfect and indelible ink, we may easily conceive that much time, much patience, and great researches, were requisite; but the author has been rewarded for all his labor, and has ultimately obtained what he has so long sought for.

Article IV.

*Discovery of an Ink which resists Chemical Agents.*—The author announces the properties of the ink which forms the subject of the fourth article, in the following terms. The ink of my composition, he says, is founded on principles different from those of common inks. It neither contains galls, Brazil nor log-wood, gum, or any preparation of iron; it is purely vegetable; it resists the action of the strongest acids, of the most concentrated alkaline solutions, and finally all solvents whatsoever.

Nitric acid has little effect on the writing made with this ink. After the action of oxymuriatic acid, the caustic alkaline solutions reduce it to the color of carburet of iron; the characters, however, remain without alteration. The principles of which it is composed, warrant its being incorruptible, and that it will retain its qualities for several years.

Such remarkable properties deserved to be proved; we have, therefore, not only subjected the ink in question, but also the writings made with it, to different trials. The results which we have obtained having been conformable to those announced by the author, we think we are warranted in considering his ink as one of the best inks to which the name of indelible has been given. At the same time we must state, that it has one of the defects which most of the indelible inks have, that of forming pretty quickly in the bottles and ink-stands a deposit which deprives the liquor above of the qualities it before had. This fault certainly would disappear, if, every time that it is used, it was shaken; but is it to be expected that this precaution will be always taken; and, as the least negligence in this respect would occasion an inconvenience, should not the author endeavor to find some method for preventing it?

Private motives having determined M. Tarry from publishing lishing the receipt of his indelible ink, we feel ourselves also dispensed from the obligation of mentioning the experiments we have made with a view of discovering the substances he has employed. We think we ought, after this, to terminate our report by inviting the class to show its satisfaction to the author for his zeal in pursuing a work which promises to be of great utility to society, by introducing an ink, which, not being susceptible of being destroyed by any chemical agent yet known, will effectually prevent the too frequent practice of altering papers, &c. now often the case.—*Annales de Chimie.*

---

### *An extraordinary Instance of Trance.*

Jussoodanundun Muhapater, one of the principal landholders of this district (Mednepoor), being required to attend the Zillah court, was reported, when called upon, to be asleep, and unable to make his appearance. A man in court hearing this excuse, observed, "Oh! if that be the case, it will be some days before he awakes." The curiosity of Mr. Rees, the judge and magistrate, being excited by the answers he received to his questions upon the subject, he proposed to me a visit to the man, with a view to inquire into this extraordinary circumstance, and afford any relief that might be required. We accordingly went to the man's house that afternoon.

We found Jussoodanundun upon a bed in his dormitory, in sound sleep, surrounded by a number of friends and relations. His pulse and breathing were scarcely perceptible, and in this state he remained two days and a half, and two nights, without motion, without taking any sustenance, or performing any of the animal functions. He was bled, but it was with difficulty that about ten ounces of blood were procured; various external stimuli were employed, and an emetic administered. By this means his pulse was considerably increased in strength, and his breathing became more perceptible; and once the stimulus applied to the nose occasioned sneezing, but he still continued in a sound sleep. Having directed a repetition of the emetic, the first producing no effect, we left him, expecting that he would soon be disturbed by its operation; this, however, was not the case, for though he vomited three times, it occasioned no interruption to his slumber.

The next morning the usual symptoms of returning animation, namely, great heat of the legs and feet, were observed; and, as his attendants said he would probably awake about twelve o'clock, no further endeavors to rouse him were employed. About twelve, as was predicted, he awoke as from a common sleep, with his usual unconsciousness of the lapse of time, having slept three nights and three days and a half.

Jussoodanundun Muhapater is a man nearly fifty years of age, strongly made, and corpulent. He relates, that about the age of one or two and thirty, he first became affected in this extraordinary manner, without being able ever to conjecture from what cause it originated. During thirteen years these fits of sleep continued seven, and sometimes eight days, with seldom more than ten or twelve days interval; for the last four years the periods of sleep have decreased to four, and rarely exceed five, days. He states that, during these fits, he has never dreamt, or been conscious of the slightest degree of animation.

The common methods of disturbing sleep have constantly, and ineffectually been resorted to; such as tumbling him about, shouting, &c. and a gun has been fired close to his ear, without producing the desired effect.

At the termination of the sleep, he rises wholly unconscious of having passed more than a common night's rest; and the only inconvenience he experiences, in consequence, is a great degree of lassitude the following day.

His general health is good, and he enjoys ordinary rest at nights, during what may be called his interval of watching.

As the shortness of his neck, and corpulency, would lead one to suppose there was a tendency to apoplexy in his case, it was surprising that, in the commencement of these fits of sleep, and for several years afterwards, his habit and appearance afforded no such indication.—Signed J. SAVAGE, Assist. Surgeon. (*Asiatic Annual Register*, vol. xi.)

*March* 14, 1809.

